# Gastric emptying scintigraphy results may influence the selection of the type of bariatric surgery: A cohort study

**DOI:** 10.1097/MD.0000000000017205

**Published:** 2019-10-11

**Authors:** Neeraj Khiyani, Mark Tulchinsky, Sana Hava, Truong An Ho, Simin Dadparvar

**Affiliations:** a Division of Nuclear Medicine and Molecular Imaging, Department of Radiology, Temple University Health System, Philadelphia; b Department of Radiology, Section of Nuclear Medicine, Milton S. Hershey Medical Center, Hershey, PA.

**Keywords:** clinical outcomes, gastric emptying scintigraphy, gastric motility, roux-en-y gastric bypass, sleeve gastrectomy

## Abstract

Bariatric surgery (BAS) may result in adverse outcomes that include appearance of gastrointestinal (GI) symptoms and/or failure to reach the weight reduction goal. This retrospective study tested the hypothesis that pre-operative gastric emptying (GE) abnormality is responsible for adverse post-surgical outcomes.

Pre-operative GE was performed using the standard solid-meal GE scintigraphy (GES) in 111 consecutive patients (105 females and 6 males, mean age 46.2 years, range 20–70 years) who were evaluated for BAS. All underwent BAS – 93 had laparoscopic sleeve gastrectomy (LSG) and 18 had Roux-en-Y (ReY) gastric bypass. All had short-term (3-6 months) and long-term (up to 54 months) follow-up with review of symptoms, physical, and laboratory examinations. Chi-square analysis was performed. *P*-value < .05 was considered significant.

Of the 111 patients, 83 had normal and 28 had abnormal pre-op GES. Sixty-eight were asymptomatic and 43 were symptomatic prior to surgery. Following surgery, 81 patients were asymptomatic and 30 were symptomatic at long-term follow-up. There was no significant difference between pre-op GE results and post-surgical adverse clinical outcome (p = ns). However, GES results seem to have guided the selection of surgical procedure significantly (*P* = .008).

Pre-operative GE study was not a strong predictor of clinical outcome in BAS. Although, it influenced the type of surgery, as when the GES was abnormal, the patient was more likely to undergo ReY and when GES was normal, they favored LSG. Interestingly, many of our symptomatic patients at 6 months post-op were asymptomatic after long-term follow-up.

## 1 Introduction

The worldwide incidence of morbid obesity is increasing and has been found to be associated with many adverse health effects and early mortality.^[1–4]^ These adverse effects include mortality and morbidity from diabetes mellitus, heart disease, gastroesophageal reflux disease (GERD), and many others.^[4]^ In the last few decades, bariatric surgery (BAS) has established itself as a definitive treatment for morbid obesity and its related complications.^[3,4]^ The two most common procedures performed are laparoscopic sleeve gastrectomy (LSG) and Roux-en-Y (ReY) gastric bypass.^[5–7]^ Randomized control trials have demonstrated that both have good rates of success and overall similar outcomes.^[3,5]^ However, recently, there has been a shift from ReY to LSG as treatment of choice for severely obese patients because of its relative ease of operation and lower complication profile.^[5,6,8]^

LSG and ReY have generally the same complications: anastomotic leak, fistula, postoperative hemorrhage, small bowel obstruction, nutritional/metabolic complications, and GERD.^[9]^ One of the most common complaints between the two surgeries is nausea and vomiting.^[7,10]^ When these complaints persist, it is necessary to evaluate for problems such as stricture, small bowel obstruction, behavioral disordered eating, or gastric motility issues.^[10,11]^ Gastric emptying scintigraphy (GES) is a standardized, noninvasive, quantitative study that evaluates gastric motor function.^[12,13]^ Gastric emptying study is currently considered to be the most reliable method to diagnosis gastric dysmotility.^[13]^ It is often used to study patients that complain about symptoms such as early satiety, epigastric pain, bloating, nausea, and vomiting.^[14]^ There are no standardized values for gastric scintigraphy for patient's after BAS and these patients are compared to values used in normal patients.^[12]^

Each gastric bypass procedure has a different motility issue associated with it; ReY and LSG may result in an increase rather than decrease in GE.^[10,15]^ In fact, there is some evidence that gastric bypass surgery may be helpful in situations of gastroparesis refractory to medical treatment.^[11,16]^ Normally, during GE, the solid meal moves from the fundus to the antrum of the stomach.^[17]^ Many radionuclide studies have demonstrated accelerated gastric motility in patients after LSG for both solids and liquids.^[18,19]^ This acceleration in motility may be due to changes in the fundus’ ability to receive and adapt to ingested food, known as fundic accommodation, and has been associated with LSG.^[20,21]^

In our institution, it was noted in the clinical follow-up of some patients that they had symptomatology of motility disorder after BAS. The decision was made for patients to undergo pre-BAS GES to predict the outcome of these patients’ post-surgery. The goal of this study was to assess whether pre-operative GES would be able to predict the outcome of patients undergoing gastric bypass surgery at short-term or long-term follow-up. We retrospectively analyzed the pre-operative GES with patients short- and long-term clinical follow-up in our institution.

## 2 Methods

Following approval by institutional Review Board, patients’ clinical history and physical examination pre- and post-BAS with GES study results were compared. Most patients were often seen by the BAS team for more than 2 years after their procedure. Patients who were doing well and asymptomatic with resolution of any surgical complications were not required to continue. Follow-up data was collected retrospectively from January 2016 to December 2018 in a large, academic healthcare system.

All patients seeking BAS were referred for pre-operative GES. One hundred and eleven consecutive patients (105 female, 6 male) with an age range of 20 to 70 years (mean age of 46.2 years) were referred. All patients that underwent GES received a formal questionnaire before and immediately after the study which evaluated symptomatology. The symptoms analyzed were dysphagia, early satiety, nausea, vomiting, abdominal discomfort, constipation, and epigastric pain. Pre-operative nuclear medicine GES results and comorbidities such as diabetes mellitus, hypertension, gastroparesis, *Heliobacter pylori*, GERD, and others were noted. The patient's postoperative motility symptoms such as nausea, vomiting, dysphagia, diarrhea, epigastric pain, and abdominal discomfort were compiled from the form bariatric surgeons notes in the electronic medical record. When symptoms were present, they were managed with appropriate therapy. Patient demographics with symptoms are summarized in Table 1.

**Table 1:**
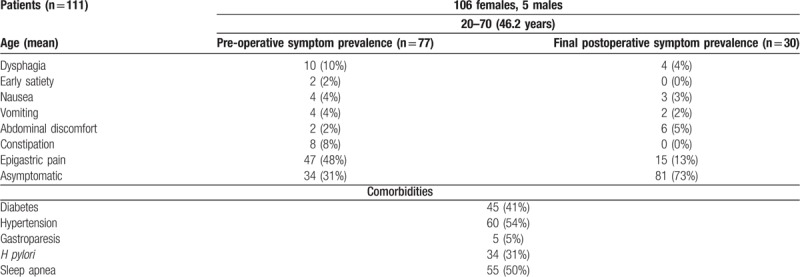
Patient demographics.

In our patient group, there were no operative modifications performed in either surgery type. Postoperative complications, including bleeding, infection, and dehiscence, were reported. Records were reviewed up to their most recent visit at the gastrointestinal and bariatric clinics. Presence or absence of motility symptoms were recorded at the end of each follow-up visit. All data were collected and analyzed. Twenty-one symptomatic patients after surgery underwent GES studies for further evaluation.

Solid-meal GES was performed using the 4-h protocol of Tougas et al.^[22]^ Patients were scheduled for solid GE prior to BAS. Patients were NPO after midnight and refrained from narcotic analgesics, anticholinergic agents, and prokinetic agents. Their blood sugar was recorded. One hundred twenty grams (4 ounces) of liquid egg was mixed with 500 μCi of technetium-99m (Tc99m) sulfur colloid. It is cooked until it becomes scrambled egg. The scrambled egg is then added to the two pieces of toasted bread with 2 packages of 1/2 ounce jelly spread on it. Patients were asked to consume the egg sandwich 350 cal, followed by 300 mL of water, within ten minutes; the time required to consume the meal was documented. If patients were unable to swallow solids or had an egg allergy, they were given 250 mL of Ensure Plus drink, with 360 cal per serving, mixed with 500 μCi of Tc99m sulfur colloid followed by 300 mL of water.^[23]^ All GE studies were conducted using the Tougas meal except for two where Ensure Plus drink had to be used instead. The patients who underwent postoperative GES, the meal amount was reduced by 50% because of the reduced gastric capacity inherent in BAS.

Immediately after meal consumption, anterior and posterior 60 s images of the abdomen were acquired with patient standing. First set of images is labeled as time point 0. Imaging occurs again at 30, 60, 90, 120, 180, and 240 min. For each time frame, the geometric mean count from the anterior and posterior images with decay correction was calculated. The main parameter measured was percent GE. Gastric emptying curves were generated using a power exponential model. All data are expressed as a percentage of the isotope emptied from the stomach. After the geometric processing, fundic accommodation at time zero and the remaining percent activity in the stomach at 2- and 4-h post-ingestion were reported. Normal GES values reported as <60% remaining at 2 h and <10% remaining at 4 h. Rapid emptying is defined as <35% remaining at 1 h. All studies were interpreted by three expert nuclear medicine physicians independently.

Patients are described as diabetic using the criteria established by the American Diabetes Association.^[24]^ The presence or absence of glycemic outcomes were determined using the standards established by the American Society of Metabolic and Bariatric surgery (ASBS).^[25]^ Remission is described as glycosylated hemoglobin <6% and the absence of anti-diabetic medications.

## 3 Statistical analysis

Contingency tables were created to evaluate for a relationship between GES result and symptomatology at short interval and final follow-up, GES result and presence of type 2 diabetes, and GES result and surgery type. The dichotomized data was evaluated by the two-tailed chi-square statistical analysis. *P*-Value was generated using the chi-square distribution. Significance level was set at < .05, corresponding to a confidence level of 95%. The contiguous data was expressed as average ± standard deviation, which was followed by median and/or ranges in parenthesis when appropriate. The calculations were performed using Microsoft Excel software.

## 4 Results

Pre-operative symptoms, postoperative symptoms, and comorbidities are summarized in Table 1. All patients underwent BAS irrespective of GES findings. One patient experienced postoperative bleeding and recovered after a short inpatient management. There were no other immediate postoperative complications, including infections or wound dehiscence. Of all patients that underwent surgery with ReY, 3 (16%) were found to have a marginal ulcer. Short-term interval follow-up clinic visits were obtained on all patients within 3 to 6 (median of 4) months. Long-term follow-up was obtained 27 ± 5 (X-54) months postoperatively in all patients.

Of the 111 patients studied, the average initial weight of 119.5 ± 20.8 kg and initial BMI was 44.8 ± 6.6 kg/m^2^. At final follow-up, the average weight was 87.5 ± 16.8 kg with an average BMI of 33.0 ± 5.7. There was an average change in BMI of 11.6 ± 5.6% and total weight loss of 26.3 ± 10.5%. The average glycosylated hemoglobin (Hb_A1c_) of the 111 patients was 6.15 ± 1.1 prior to BAS. After surgery, the average Hb_A1c_ was 5.7 ± 0.86.

Of all the GES studies analyzed, the average percentage of the meal remaining was 38.8 ± 16.9% (range 2–71%) at 2 h and 7.1 ± 7.8% (range 1–78%) at 4 h. Normal GE was found in 83 patients (74.8%) and abnormal in 28 (25.2%). Of the 28 abnormal pre-operative GES studies, the average meal percentage remaining after consumption of the radiolabeled meal was 58.6 ± 14% (range 29–77%) at 2 h and 18 ± 8% (range 2–38%) at 4 h. All patients’ fundic accommodations were adequate.

At short-interval follow-up, 67 (60.4%) patients were asymptomatic while 44 (39.6%) were symptomatic. There was no significant difference between pre-op GE results and short-interval adverse clinical outcomes (χ^2^ = 0.748; *P* = .38). At long-term follow-up, 76 (68.5%) patients were asymptomatic while 35 (31.5%) remained symptomatic. Of the 111 patients, 21 (18.9%) suffered postoperative symptoms severe enough to warrant a postoperative GES study. All 21 studies demonstrated rapid GE with inadequate fundic accommodations. The average meal percentage remaining after consumption of the radiolabeled meal was 9.8 ± 5.4% (range 0–22%) at 1 h.

**Figure 1:**
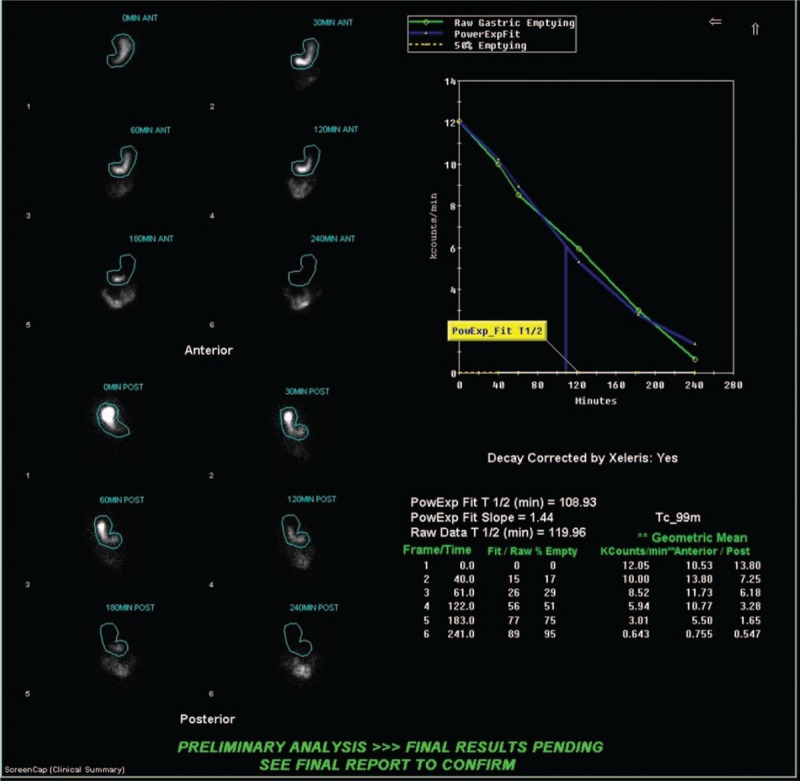
Normal gastric emptying scintigraphy in a 58-year-old woman who underwent LSG. Pre-op GES: normal fundic accommodation; at 2 h: 49%, N < 60% and at 4 h: 5%, N < 10%. Patient had a normal postoperative outcome.

Ninety-three patients underwent LSG (Fig. 1). Of this patient group, 74 (79.6%) had normal pre-operative GES results and 19 (20.4%) had abnormal results. At short-interval follow-up after surgery, 59 (63.4%) patients were asymptomatic, while 34 (36.6%) were symptomatic (Table 2). At long-term follow-up postoperatively, 71 (76.3%) patients had no symptoms while 22 (23.7%) did.

**Table 2:**
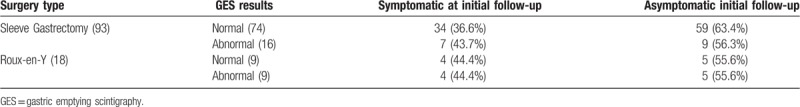
Pre-op gastric emptying results and postoperative outcomes based on type of surgery.

**Figure 2:**
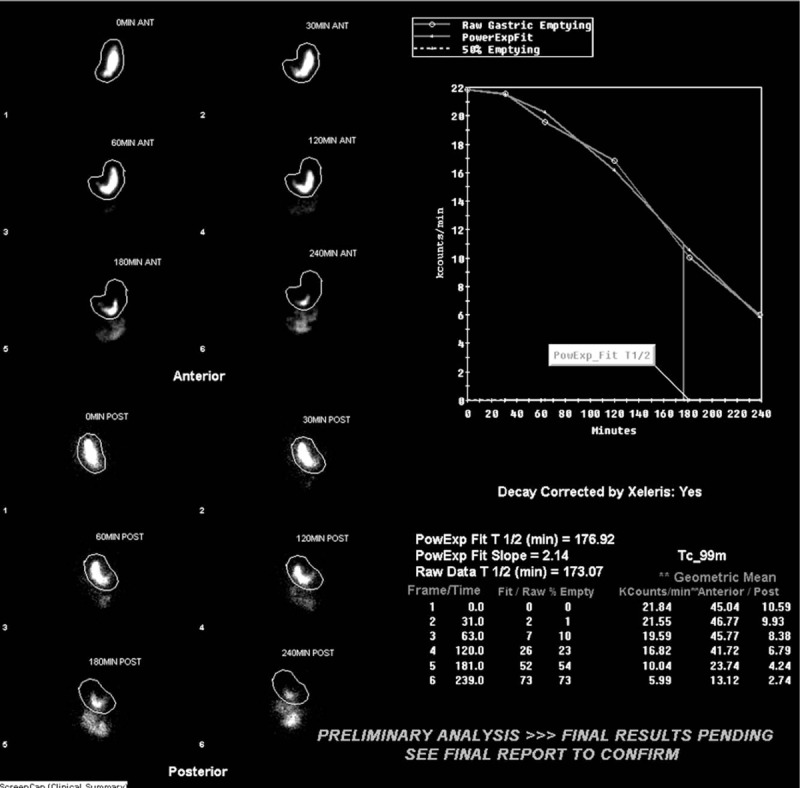
Delayed gastric emptying scintigraphy in a 55-year-old woman who underwent ReY. Pre-op GES: normal fundic accommodation; at 2 h remaining 77%, N < 60% and at 4 h remaining 27%, N < 10%. Post op: She complained of abdominal pain on short- and long-term follow-up.

Eighteen patients underwent ReY procedure (Fig. 2). Nine (50%) patients had normal pre-operative GES studies; 9 (50%) patients had abnormal studies. Compared to the LSG group, the patients undergoing ReY had significantly more abnormal GES studies.

There was no significant correlation of pre-op GES study results and post-surgical clinical outcome (χ^2^ = 0.911; *P* = .37) among the two groups. As surgery type and GES result were compared (Table 3), when GES was abnormal, the surgeons preferred ReY. However, when GES was normal, LSG was favored. The chi-squared statistic is 6.991. The result was significant with *P*-value is .008.

**Table 3:**
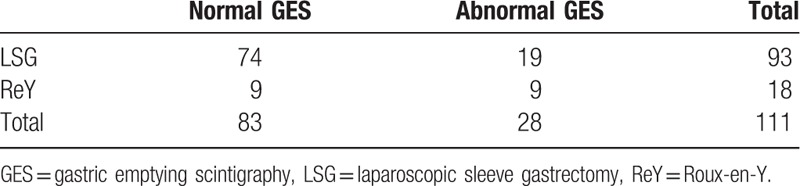
Surgery type and GES results.

Comparison was made between patient symptoms in early and late post-surgical period (Table 4). Of the 93 patients that underwent LSG, 34 (36.6%) were symptomatic at initial follow-up, but only 22 (23.7%) were symptomatic at late follow-up. In comparison, 8 (44.4%) of the 18 patients that underwent ReY were found to have symptoms at initial and delayed follow-up.

**Table 4:**
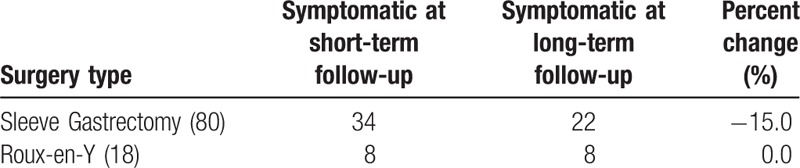
Percent of improvement in symptomatic patients based on surgery type.

## 5 Discussion

At short interval postoperative follow-up, 67 (60.4%) patients were found to be asymptomatic (52/15 with normal/abnormal GES studies). Forty-four (39.6%) patients had motility symptoms (29/15 with normal/abnormal GES studies) which included diarrhea, epigastric pain, dysphagia, vomiting, and abdominal pain.

Overall, 81 patients were asymptomatic (63/18 with normal/abnormal GES studies) at the end of long-term follow-up. The remaining 30 patients (20/10 with normal/abnormal GES studies) had post-prandial symptoms postoperatively in 22 patients after LSG and 8 after ReY. When comparing reported gastric motility symptomatology before and after surgery, most patients experience alleviation of their symptoms by the end of follow-up. All 26 patients without pre-operative symptoms (59%) had negative GES. However, 43 patients (39%) with gastric motility symptoms had a prior negative GES study.

Our data agrees with Burgerhart et al who reported their patients experienced more post-prandial symptoms after LSG.^[20]^ The reason for the increased symptom perception may be due to increased visceral hypersensitivity as a result of the procedure as reported by Farré et al.^[26]^ In addition, Burgerhart et al found that abnormal GES after surgery did not correlate with symptomatology, but patients after LSG were found to have increased rate of GE.^[20]^ In our cohort, twenty-one patients continued to have symptoms that were evaluated with a post-surgical GES and demonstrated an increased GE rate with inadequate fundic accommodation regardless of their symptomatology. When LSG is performed, the fundus of the stomach is removed and there is alteration of the compliance and contractility of the gastric sleeve resulting in disruption of the neurohormonal pathways that controls motility and rapid emptying occurs.^[18,27]^ Interestingly, there were no observable changes in gastric retention despite significant improvements in weight and HbA1c.

In our patient cohort, the most common symptom complaint was GERD at short-term follow-up. Forty-eight patients complained of GERD symptoms at their short-term follow-up (38 underwent LSG and 10 ReY) and 15 at their last follow-up (10 underwent LSG while the rest underwent ReY). Dupree et al reported in a large patient population that LSG did not reliably improve GERD-like symptoms and may even induce these findings in previously asymptomatic patients. On the other hand, ReY was found to improve GERD symptoms. However, we did not experience the same findings in our patients undergoing either types of surgery.^[4]^ His study only analyzed patients for six months postoperatively. We followed our patients for up to 2 years and observed improvement in GERD symptoms regardless of surgery type.

Diabetes mellitus is known to impact gastric motility. Forty-five (40.6%) patients were diabetic or pre-diabetic. Only 13 of the diabetic patients had abnormal pre-operative GES studies. Interestingly, of the 66 (59.4%) patients without a history of diabetes mellitus, 20 had a delayed pre-operative study, while the other patients had normal GES studies. The presence of diabetes did not seem to impact pre-operative GES results significantly (*P* = .32). By the end of the long-term follow-up period, 19 of patients with pre-operative diabetes had complete remission of their symptoms as defined by the American Society of Bariatric Surgery.

Finally, GES results seemed to have guided the selection of surgical procedure. Patients with abnormal GES findings were significantly more likely to have undergone ReY than patients with normal GES findings. Like Salminen et al, it can be extrapolated that abnormal GES increased the likelihood for referral to ReY while normal GES predisposed to referral for LSG. If patients demonstrated abnormal motility on imaging, it would be logical to opt for a procedure that left as little pathologic gastric tissue as possible remaining (Fig. 2). This could be achieved with ReY.^[8]^ However, if gastric motility shown to be normal, removing as little tissue as possible to achieve greatest bariatric results would be prudent (Fig. 1).

This study is limited due to retrospective analysis of a small patient population that underwent BAS with pre-operative GES at our institution. In addition, given the discrepancy in sample size between LSG and ReY, there is limited comparison between these two groups. This is because LSG has been demonstrated to have better outcomes than ReY. As a result, sleeve gastrectomy procedures are more often performed.^[5]^ Another limitation is that our patients were not monitored daily. Though our patient population had lengthy follow-up, a prospective study with a larger patient population is recommended which could further elucidate a relationship between pre-operative GES results and long-term outcomes.

## 6 Conclusion

To our surprise, pre-operative GES could not predict the outcome of patients undergoing gastric bypass surgery at short- and long-term follow-up. Clinical outcomes did not correlate with prior history of any other motility disorder or diabetes. Patients suffering from motility symptoms prior to BAS with ReY and LSG cannot be expected to remain symptomatic after surgery. While, patients who are asymptomatic prior to surgery can suffer from motility disorder post-op. Interestingly, many of our symptomatic patients at 6 months post-op were asymptomatic after long-term follow-up. The GES prior to surgery was significant in identifying the right procedure for the patient with abnormal motility that can benefit from ReY. Sleeve gastrectomy leaves more remaining gastric function than Rey procedure. Therefore, it would be conceptually appropriate for patients with abnormal GES to undergo ReY instead of LSG. We recommend more investigation in selection of type of surgery since, as it stands now; LSG is more commonly performed by bariatric surgeons.

## Acknowledgments

None

## Author contributions

**Conceptualization:** Sana Hava, Truong An Ho, Simin Dadparvar.

**Data curation:** Neeraj Khiyani, Sana Hava, Truong An Ho.

**Formal analysis:** Mark Tulchinsky, Sana Hava, Simin Dadparvar.

**Funding acquisition:** Simin Dadparvar.

**Investigation:** Neeraj Khiyani, Sana Hava, Truong An Ho, Simin Dadparvar.

**Methodology:** Neeraj Khiyani, Mark Tulchinsky, Sana Hava, Truong An Ho, Simin Dadparvar.

**Validation:** Mark Tulchinsky.

**Writing – original draft:** Neeraj Khiyani, Mark Tulchinsky, Simin Dadparvar.

**Writing – review & editing:** Neeraj Khiyani, Mark Tulchinsky, Simin Dadparvar.

Simin Dadparvar orcid: 0000-0002-4700-3060.
